# Effect of *Porphyromonas gingivalis* infection on gut dysbiosis and resultant arthritis exacerbation in mouse model

**DOI:** 10.1186/s13075-020-02348-z

**Published:** 2020-10-19

**Authors:** Yuta Hamamoto, Kazuhisa Ouhara, Syuichi Munenaga, Mikio Shoji, Tatsuhiko Ozawa, Jyunzo Hisatsune, Isamu Kado, Mikihito Kajiya, Shinji Matsuda, Toshihisa Kawai, Noriyoshi Mizuno, Tsuyoshi Fujita, Shintaro Hirata, Kotaro Tanimoto, Koji Nakayama, Hiroyuki Kishi, Eiji Sugiyama, Hidemi Kurihara

**Affiliations:** 1 Department of Periodontal Medicine, Division of Applied Life Sciences, Institute of Biomedical & Health Sciences, Hiroshima University, 1-2-3 Kasumi, Minami-ku, Hiroshima, 734-8553 Japan; 2 Department of Microbiology and Oral Infection, Graduate School of Biomedical Sciences, Nagasaki University, 1-7-1 Sakamoto, Nagasaki, 852-8588 Japan; 3 Department of Immunology, Faculty of Medicine, Academic Assembly, University of Toyama, 2630 Sugitani, Toyama, 930-0194 Japan; 4 Antimicrobial Resistance Research Center, National Institute of Infectious Diseases (NIID), Toyama 1-23-1, Shinjuku-ku, Tokyo, 162-8640 Japan; 5 Department of Orthodontics and Craniofacial Developmental Biology, Graduate School of Biomedical & Sciences, Hiroshima University, 1-2-3 Kasumi, Minami-ku, Hiroshima, 734-8553 Japan; 6 Department of Periodontology, Nova Southeastern University College of Dental Medicine, 3200 South University Drive, Fort Lauderdale, FL 33328 USA; 7 Department of Clinical Immunology and Rheumatology, Hiroshima University Hospital, 1-2-3 Kasumi, Minami-ku, Hiroshima, 734-8551 Japan

**Keywords:** *Porphyromonas gingivalis*, Rheumatoid arthritis, Periodontitis, Citrullinated protein, Dysbiosis

## Abstract

**Background:**

*Porphyromonas gingivalis* (Pg) infection causes periodontal disease and exacerbates rheumatoid arthritis (RA). It is reported that inoculation of periodontopathogenic bacteria (i.e., Pg) can alter gut microbiota composition in the animal models. Gut microbiota dysbiosis in human has shown strong associations with systemic diseases, including RA, diabetes mellitus, and inflammatory bowel disease. Therefore, this study investigated dysbiosis-mediated arthritis by Pg oral inoculation in an experimental arthritis model mouse.

**Methods:**

Pg inoculation in the oral cavity twice a week for 6 weeks was performed to induce periodontitis in SKG mice. Concomitantly, a single intraperitoneal (i.p.) injection of laminarin (LA) was administered to induce experimental arthritis (Pg-LA mouse). Citrullinated protein (CP) and IL-6 levels in serum as well as periodontal, intestinal, and joint tissues were measured by ELISA. Gut microbiota composition was determined by pyrosequencing the 16 s ribosomal RNA genes after DNA purification of mouse feces. Fecal microbiota transplantation (FMT) was performed by transferring Pg-LA-derived feces to normal SKG mice. The effects of Pg peptidylarginine deiminase (PgPAD) on the level of citrullinated proteins and arthritis progression were determined using a PgPAD knockout mutant.

**Results:**

Periodontal alveolar bone loss and IL-6 in gingival tissue were induced by Pg oral infection, as well as severe joint destruction, increased arthritis scores (AS), and both IL-6 and CP productions in serum, joint, and intestinal tissues. Distribution of Deferribacteres and S24-7 was decreased, while CP was significantly increased in gingiva, joint, and intestinal tissues of Pg-inoculated experimental arthritis mice compared to experimental arthritis mice without Pg inoculation. Further, FMT from Pg-inoculated experimental arthritis mice reproduced donor gut microbiota and resulted in severe joint destruction with increased IL-6 and CP production in joint and intestinal tissues. The average AS of FMT from Pg-inoculated experimental arthritis was much higher than that of donor mouse. However, inoculation of the PgPAD knockout mutant inhibited the elevation of arthritis scores and ACPA level in serum and reduced CP amount in gingival, joint, and intestinal tissues compared to Pg wild-type inoculation.

**Conclusion:**

Pg oral infection affected gut microbiota dysbiosis and joint destruction via increased CP generation.

## Background

Periodontal disease (PD) is a highly prevalent infectious disease caused by periodontopathogenic bacteria, such as *Porphyromonas gingivalis* (Pg), *Tannerella forsythia*, and *Treponema denticola*, called red complex [1]. Host immune response against periodontopathogenic bacterial challenge causes severe destruction of dental supportive tissues, including cementum, periodontal ligament, and alveolar bone [2]. More specifically, local and systemic elevation of inflammatory cytokines, interleukin (IL)-6 and TNF-α, affect PD progression through T cell activation [3]. Several studies have shown a strong correlation between PD, especially in the presence of Pg infection, and systemic disease, including non-alcoholic steatohepatitis (NASH), rheumatoid arthritis (RA), pre-birth, low weight birth, Alzheimer’s disease, and Buerger disease [4–8].

RA is a systemic inflammatory autoimmune disease characterized by chronic inflammation and joint tissue destruction, potentially leading to functional disability [9]. The cause of RA is not fully understood, but a multifactorial pathogenesis, involving both environmental and genetic factors, has been widely accepted [10, 11]. Among the proposed environmental triggers, PD is currently considered as a risk factor for RA and may play a central role in disease initiation [12]. Furthermore, Pg, a Gram-negative anaerobic bacterium, has an important role in the pathogenic hypothesis linking RA and PD, owing to the production of peptidylarginine deiminase (PAD) and the strong protease gingipain [13]. PAD derived from Pg (PgPAD) can generate citrullinated proteins (CP), in a manner similar to that of endogenous PAD (PADI4), which might result in the production of anti-*citrullinated* protein/peptide antibody (ACPA) [14].

We previously showed that systemic or oral Pg administration exacerbated RA-like experimental arthritis in a mouse model [15, 16]. Specifically, with systemic Pg infection, bacterial proteins were detected in joint tissues showing arthritis outcomes [15]. However, these bacteria-derived proteins were not recovered from the affected arthritis joints in the Pg oral administration model [16], likely because the pathogenic effect of Pg in arthritis progression does not occur through direct bacterial stimulation. Therefore, there is a possibility that oral infection of Pg may impact on pathogenic outcomes of arthritis indirectly, possibly via altering gut microbiome which was recently found to have a remarkable effect on the manifestations of a number of systemic diseases [17, 18], including arthritis [19, 20]. Indeed, Kato et al. reported that oral Pg administration can alter the gut microbiome [21].

Dysbiosis of gut microbiota was observed in RA patients, and alterations in the microbiome could distinguish RA patients from healthy individuals [22]. Clustering analysis at the genus level showed 4 different type of clusters. Each cluster was characterized by a high abundance of the following genera: Ruminococcus in cluster 1, Bacteroides in cluster 2, Blautia and Faecalibacterium in cluster 3, and Prevotella in cluster 4. Cluster 4, which was characterized by a high abundance of Prevotella, consisted of only the feces from RA patients. Changing in the amount and type of oral microbiota was also reported in RA patients [23]. Especially, an increased abundance of *Prevotella*, *Aggregatibacter*, *Treponema*, and *Veillonella* was reported. Furthermore, gut dysbiosis by oral inoculation of Pg was shown to contribute to the progression of experimental arthritis [22]. Periodontopathogenic bacteria, including Pg, exists in dental plaque (10^10^ [colony-forming units (CFU)]/g) and the daily inoculation of 1–2 L saliva (10^9^ CFU/mL) [24]. Therefore, it is plausible that swallowing mass of dental plaque and saliva with Pg can affect the composition of gut microbiota. To support this hypothesis, inoculated Pg can also survive in the acid conditions of gastric juice [25]. Previous studies showed that Pg could exacerbate the pathologic outcomes of experimental arthritis [15]. The mechanisms of Pg-induced arthritis included variant immune response in T cells, activation of osteoclastogenesis in joint tissues, systemic and local C5a elevation, and change in gut microbiota [16]. In humans, serum ACPA is used as a marker for RA diagnosis. Immune complex consisting of ACPA and CP is also an important molecule for joint destruction. Therefore, monitoring CP generation and ACPA synthesis may be good indicators of RA onset [11, 26]. Of animal models used in RA studies, the SKG mouse was the only model for which reported elevated ACPA during progression of experimental arthritis was reported [27]. Taken together, these immune responses may be elicited after Pg inoculation and gut dysbiosis. Therefore, this study used the SKG model to investigate the effect of Pg inoculation on the progression of experimental arthritis, including an analysis of changes in gut microbiota and CP generation in serum and gingival, intestinal, and joint tissues.

## Methods

### Preparation of bacteria

Bacteria used in this study were purchased from ATCC. Pg W83 and 33277 were cultured on a sheep blood agar plate using the Anaeropack system (Mitsubishi Gas Chemical, Tokyo, Japan) at 37 °C. After a 2-day incubation, Pg was inoculated in 40 mL of trypticase soy broth supplemented with 1% yeast extract, hemin (200 μg), and menadione (20 μg). Bacteria were harvested in the exponential growth phase and washed with phosphate buffered saline (PBS) for experiments.

### Construction of Pg knockout mutant

The Pg *pad* knockout mutant was constructed by double recombination of the target gene and *ermF* introduction, as previously described [28]. The targeting DNA was constructed as follows. The 0.5 kb-upstream and 0.5 kb-downstream regions of the *pad* gene were amplified with two pairs of primers (pad-Up-F: [GGTCTCACACGAGAGGATACTATGGTCTAT]/pad-Up-R: [CGGGGGATCCTGTTTGATATGTTTTATGAT]; pad-Dw-F: [TAGGGGATCCGGGGCCTTATTTGAGAATAC]/pad-Dw-R: [AGCAGAGGTTACGAGCTTAACCAGAGATGC], where ‘Up,’ ‘Dw,’ ‘F,’ and ‘R’ indicate upstream, downstream, forward, and reverse, respectively) using the genome of ATCC 33277 as a template. The *ermF* region in the *ermF* DNA cassette was amplified with ermF-F: [ATATCAAACAGGATCCCCCGATAGCTTCCG]/ermF-R: [ATAAGGCCCCGGATCCCCTACGAAGGATGA] using the genome of *gtfF* (PGN_1668)::*ermF* mutant (KDP611) as a template [29]. Using the three purified products, PCR was performed with pad-Up-F/ pad-Dw-R. Finally, the desired PCR product was purified and introduced into Pg ATCC 33277 by electroporation. Transformants were selected on blood agar plates containing 10 μg/mL erythromycin. The mutation of target gene was confirmed by PCR using pad-Up-R/pad-Dw-R. Correct *ermF* gene insertion of Pg *pad* knockout mutant was also verified by sequencing.

### Generation of Pg-infected experimental arthritis mouse model

All animal experimental procedures employed in this study were approved by the Ethics Committee of Hiroshima University (approval No. A16-33) and performed as previously reported [16]. Briefly, SKG mice were maintained under specific pathogen free (SPF) condition until inoculation of bacteria and feces. The food used in this study was a regular chow for mouse with gamma irradiation (Type MF; Oriental Yeast Co., Ltd., Tokyo, Japan). Laminarin derived from *Laminaria digitata *(LA) was purchased from Sigma-Aldrich (L9634, St. Louis, MO, USA). LA was dissolved in PBS at 100 mg/mL. To induce experimental arthritis, LA (10 mg/100 μL/mouse) was administered to SKG mice by an intraperitoneal (i.p.) injection. Pg was also inoculated (10^8^ bacterial cells/50 μL in 2% carboxymethylcellulose (CMC) solution) twice a week for 6 weeks. LA injection and Pg inoculation were started the same day. The same volume of CMC solution was inoculated as a negative control. Mice were divided into 3–4 groups (5 or 6 mice per group) in each experiment (Ctrl: CMC inoculation, LA: LA i.p injection, Pg: Pg oral inoculation, Pg/LA: LA i.p. injection with Pg oral inoculation, LA-FMT: LA i.p. injection with FMT from the LA group, Pg/LA-FMT: LA i.p. injection with FMT from the Pg/LA group). Power calculation was performed by the same method as that reported in our previous publication that used the SKG mouse model to evaluate the effects of Pg on the induction of arthritis [16]. In the above noted publication, arthritic score of Pg+LA treated group and that of LA alone group showed 3.6 + 1.4 and 1.0 + 0.3, respectively (average + SD). Accordingly, the sample size required for the in vivo experiments, i.e., *n* = 5/group, was determined based on 80% power at 0.05 significance.

### Evaluation of alveolar bone level in mouse

The alveolar bone level (ABL) of SKG mice was evaluated by Kawai’s methods described previously [30]. Briefly, after methylene blue staining (Sigma-Aldrich) for 10 min, the upper molar jaw was washed with PBS three times. The length of the blue-stained root surface of all molar teeth from the enamel-cement junction to the top of the alveolar bone was measured. Differences between treated mice and the control group (no treatment) were evaluated.

### Clinical assessment of arthritis score (AS) in SKG mice

Joint swelling was monitored by inspection and scored as follows: 0, no joint swelling; 0.1, swelling of one finger joint; 0.5, mild swelling of the wrist or ankle; 1.0, severe swelling of the wrist or ankle. Scores for all digits, wrists, and ankles were totaled for each mouse, as reported previously [31].

### IL-6 measurement in mouse tissue

Serum and tissues from gingiva, leg joint, small intestine, and large intestine were collected from each mouse and homogenized by cool-mill (#TK-CM20S, Tokken, Inc., Chiba, Japan) in RIPA Lysis and Extraction Buffer (#89900, Thermo Fisher Scientific, Tokyo, Japan, 100 mg tissue/100 μL) with 0.1% phenylmethanesulfonyl fluoride (PMSF, Sigma-Aldrich) and 1% proteinase inhibitor cocktail (#87786, Thermo Fisher Scientific). The sera were diluted four times by PBS. Supernatants of the tissue homogenates and sera were used for IL-6 measurement by ELISA (for mouse IL-6, #431304; BioLegend Inc., San Diego, CA, USA), according to the manufacturer’s instructions. Briefly, a solid-phase anti-IL-6 monoclonal antibody (diluted in coating buffer to a final concentration of 1 μg/mL) was coated onto a 96-well ELISA plate (BD Falcon, Franklin Lakes, NJ, USA) for target capture. After blocking each well with 1% BSA in PBS supplemented with 0.05% Tween 20 (PBST), the sample or standard (diluted in PBST from 1 ng/mL to zero) was applied to each well. After application of the detection antibody (diluted in PBST to a final concentration of 1 μg/mL), horseradish peroxidase (HRP) conjugated with anti-IgG (2000-fold dilution in PBST) was applied to the wells. Colorimetric reactions were developed with o-phenylenediamine (Sigma-Aldrich) in the presence of 0.02% H_2_O_2_. Color development was stopped with H_2_SO_4_ (2 N) and measured using an ELISA reader (OD_405,_ Varioskan LUX). The actual concentration of the target was calibrated by referring to a standard curve prepared by serial dilutions. Each sample was examined in triplicate wells of a 96-well ELISA plate. The limits of mouse IL-6 detection for each analyte were 15.6 pg/mL.

### Detection of citrullinated protein in mouse tissue

Serum and homogenized tissues from the gingiva, leg joint, small intestine, and large intestine were diluted in sodium bicarbonate buffer (pH 9.4, 10 μg/mL) and coated onto a 96-well ELISA plate. After blocking each well with 1% BSA and sucrose in PBST, anti-citrulline monoclonal IgG (clone CCP-Ab1: generated from B cells isolated from an RA patient, 10 μg/mL) was reacted in PBST at room temperature for 2 h [32]. Subsequently, anti-human IgG conjugated with HRP (2000-fold dilution in PBST) was applied to the wells. Colorimetric reactions were performed by the same method as that was used for IL-6 measurement.

### Measurement of serum ACPA level

Sera from each group were collected to measure ACPA levels. Briefly, cyclic citrullinated peptides (CCP) (Orgentec, Chicago, IL, USA, diluted in PBS pH 7.2 at final concentration of 0.5 μg/mL) were pre-coated onto a 96-well ELISA plate (BD Falcon, Franklin Lakes, NJ, USA) for target capture. After blocking each well with 1% BSA in PBST, serum (4-fold dilution) or standard (diluted in PBST from 1 ng/mL to zero) was applied to each well. After application of the detection antibody (diluted in PBST to a final concentration of 1 μg/mL), anti-IgG conjugated with HRP (2000-fold dilution in PBST) was applied to the wells. Colorimetric reactions were developed using the same protocol as that was used for IL-6 detection. The actual concentration of the target was calibrated by referring to a standard curve prepared by serial dilutions. Each sample was examined in triplicate wells of a 96-well ELISA plate.

### Histological observation

Ankle joints were fixed in 4% buffered formalin and embedded in paraffin wax. Subsequently, the tissues were sliced at a thickness of 7 μm and mounted on glass slides. The paraffin-embedded sections were stained with hematoxylin and eosin (H&E). The severity of inflammation and cartilage damage was scored in reference to the published criteria [33].

### Statistical analysis

All experiments were performed at least three times independently. Data are expressed as mean ± standard deviation (SD). Statistical analyses between two groups were performed using Mann-Whitney *U* test for non-normal distribution. For multiple comparisons, the Tukey-Kramer test or Bonferroni-corrected Mann-Whitney *U* test was used. *P* < 0.05 was considered significant.

### Metagenomics of gut microbiota

Bacterial DNA was extracted from feces as described previously [34]. In brief, feces were collected from all groups of mice (Ctrl, LA, Pg, Pg/LA, LA-FMT, Pg/LA-FMT) before treatment as a control (day 0) and at the end of experiments (day 42). The bacterial composition of day 0 was confirmed the no significant difference. The collected feces were suspended in PBS (10% sodium dodecyl sulfate, 10 mM Tris-HCl, and 1 mM EDTA, pH 8.0), and the bacterial DNA was purified using the DNeasy PowerSoil Kit according to the manufacturer’s instructions (with range of total DNA from 349.8 ng/μl to 611.8 ng/μl, #12888-100 QIAGEN, Germantown, MD, USA). The concentration and quality of purified DNA was determined by Qubit® Fluorometer (Thermo Fisher Scientific, Tokyo, Japan) and gel electrophoresis. As the positive control of bacterial DNA, Pg W83 was also purified with the same protocol. In order to detect Pg DNA from the tissues of SKG mouse, DNA was purified from each tissue by GenCheck DNA purification kit (FASMAC Co., Ltd., Hokkaido, Japan). Then, 16s rRNA gene was detected by PCR as shown previously [35].

Afterwards, the V4 variable region (515F–806R) of bacterial 16S rRNA genes was amplified and used for sequencing on an Illumina Miseq as previously described [36]. Each reaction mixture contained 15 pmol of each primer (16S Amplicon PCR Forward Primer 5′-TCGTCGGCAGCGTCAGATGTGTATAAGAGACAGCCTACGGGNGGCWGCAG, 16S Amplicon PCR Reverse Primer 5′-GTCTCGTGGGCTCGGAGATGTGTATAAGAGACAGGACTACHVGGGTATCTAATCC), KAPA HiFi HotStart ReadyMix (× 2) (Hokkaido System Science Co., Ltd., Sapporo, Japan), 50 ng extracted DNA, and sterilized water to reach a final volume of 50 μL. PCR conditions were as follows: 95 °C for 3 min; 25 cycles of 95 °C for 30 s, 55 °C for 30 s, and 72 °C for 30 min; and completed by the final extension at 72 °C for 5 min. The PCR product was purified by AMPure XP (Beckman Coulter, Inc., Brea, CA, USA) and quantified using a Quant-iT PicoGreen ds DNA Assay Kit (Life Technologies Japan Ltd., Tokyo, Japan). Mixed samples were prepared by pooling approximately equal amounts of PCR amplicons from each sample. The pooled library was analyzed with an Agilent High Sensitivity DNA Kit on an Agilent 2100 Bioanalyzer (Agilent Technologies, Santa Clara, CA). Real-time PCR was performed on the pooled library using a KAPA Library Quantification Kit for Illumina, following the manufacturer’s protocols, and a sample library with a 20% denatured PhiX spike-in was sequenced by Miseq using a 600 cycles kit to obtain 2 × 250 bp paired-end reads. Taxonomic assignments and estimation of relative abundance of sequencing data were performed using the analysis pipeline of the QIIME software package [37]. An operational taxonomic unit (OTU) was defined at 97% similarity. OTUs indicating relative abundance under 0.05% were filtered to eliminate noise. The OTU was assigned a taxonomy based on a comparison with the Silva database using UCLUST [38].

### Fecal microbiota transmission (FMT)

Fresh mouse feces from the LA and Pg/LA group were collected 6 weeks after when Pg inoculation was started. Collected feces from the same group (two fecal pellets [10–30 mg/fecal pellet] from each mouse; *n* = 6/group) were pooled and homogenized in anaerobic resuspension buffer (10 mg/ml, 2% Lab–Lemco powder, 0.1% L-cysteine, 0.045% KH_2_PO_4_, 0.09% NaCl, 0.045% (NH_4_)_2_SO_4_, 0.0045% CaCl_2_, 0.0045% MgSO_4_ and 40% glycerol in 1000 mL) and kept at − 80 °C until used. Before FMT, SKG mice were treated with antibiotics (ampicillin (Sigma-Aldrich) 1 g/L, metronidazole (Sigma-Aldrich) 1 g/L, neomycin (Sigma-Aldrich) 1 g/L, vancomycin (Sigma-Aldrich) 0.5 g/L) dissolved in water for 1 week. At this point, feces were collected prior to FMT and the changing of gut microbial composition was confirmed. Female SKG mice (6–8 weeks old) received gastric intubation of 250 μL of the fecal suspensions from donor mice twice in a week. Subsequently, these mice were intraperitoneally injected with LA (100 μg) at 1 week after bacterial gastric intubation. AS and ankle thickness were monitored every week for 6 weeks after injection. Six weeks after FMT, feces of two recipient groups that received LA and Pg/LA (*n* = 5/group) along with respective FMT were collected independently and microbiota composition of the each mouse was analyzed by next generation DNA sequencing of bacterial 16 s rRNA.

### Western blotting

Homogenized tissues from the leg joint and gingival tissue were applied to a 12% SDS-polyacrylamide gel for electrophoresis and electronically transferred onto nitrocellulose membranes (BioRad Laboratories, CA, USA). The membrane was blocked with 1% non-fat dried milk at room temperature for 1 h and then reacted with anti-PADI2 rabbit monoclonal IgG (10 μg/ml, 12,110–1-AP, PROTEINTECH JAPAN, Japan), anti-PADI4 rabbit monoclonal IgG (10 μg/ml, EPR20706, abcam, Japan), or anti-Pg rat serum (1000 folds dilution) in PBST at 4 °C for 12 h. The membrane was incubated with HRP-conjugated with sheep anti-rabbit IgG or sheep anti-rat IgG in PBST at room temperature for 1 h. Immunodetection was performed according to the manual supplied with ECL Plus Western blotting reagents (GE Healthcare Life Sciences, Japan). As a control, the amount of Glyceraldehyde 3-phosphate dehydrogenase (GAPDH) was detected by anti-GAPDH antibody (10 μg/ml, HRP-60004, PROTEINTECH JAPAN, Japan) The density of target PADI2 and PADI4 bands was measured by NIH Image-J software.

## Results

### Evaluation of periodontal tissue in experimental arthritis model mice with Pg infection

**Fig. 1:**
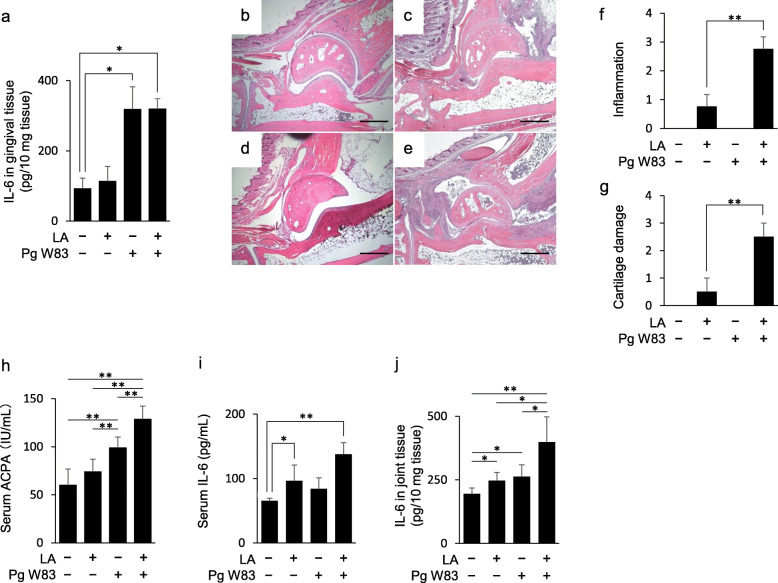
Assessment of periodontal tissue and evaluation of joint swelling in Pg-administered SKG mice. To determine the effect of Pg infection in periodontal tissue, Pg W83 (10^8^ CFU/50 mL/mouse in 2% CMC/PBS solution) was administered into the oral cavity of SKG mice (6–8 weeks old, female) twice a week for 42 days. SKG mice also received LA i.p. injection. All mice were sacrificed, and serum, gingival tissue, and leg joint tissues were collected. Increased IL-6 in gingival tissue of each group was found (**a**). Data represent mean ± SD of 6 mice per group. Ctrl, PBS inoculation; LA, PBS inoculation + LA i.p. injection (10 μg/mouse); Pg, Pg inoculation; Pg/LA, Pg inoculation + LA i.p. injection. H&E staining of joint tissue sections (**b** Ctrl, **c** LA, **d** Pg, **e** Pg+LA). Histological scores of inflammation, and cartilage damage from each group of mice (*n* = 6 per group) (**f**, **g**). Data represent the mean ± SEM. Original magnification; scale bar 500 μm. The titer of serum ACPA in mice was measured by ELISA (**h**). The amount of IL-6 in each group was determined by ELISA (**i** serum, **j** joint tissue). Data represent mean ± SD (**b**–**d** and **i**–**k**) of 6 mice per group. Statistical analyses were performed using the Tukey-Kramer test and Bonferroni corrected Mann-Whitney *U* test for multiple comparisons (*P < 0.05, ** *P* < 0.01)

Oral infection of Pg induced alveolar bone loss in the Pg and Pg/LA groups (Fig. S1a and b). However, the Ctrl and LA groups did not show any alveolar bone loss. Additionally, IL-6 production in mouse gingival tissue was measured and it was found to be increased in the Pg and Pg/LA groups compared to the Ctrl group (Ctrl 92.2 pg/10 mg tissue, Pg 318.3 pg/10 mg tissue, Pg/LA 319.4 pg/10 mg tissue). Although it is well established that RA affects periodontitis clinically, and that especially the treatment of RA resulted in the improvement of periodontitis [39], IL-6 production in mouse gingival tissue of the LA group was similar to that of the Ctrl group (Fig. 1a). In the purified DNA from serum, feces, gingival tissue, tongue, lung, stomach, small intestine, and large intestine of the Pg group, Pg-derived DNA was only detected from gingival tissue, tongue, and lung tissue (Fig. S1d).

### Assessment of experimental arthritis in mice

To assess experimental arthritis in mice with Pg oral inoculation, serum and joint tissues were examined. AS increased in the LA group 5 weeks after LA injection. Four weeks after LA injection and Pg inoculation, AS score of the Pg/LA group was also significantly elevated matching the results from our previous report [16]. However, AS of Ctrl and Pg groups did not show the induction of joint swelling (Fig. S1c). H&E-stained sections of joint tissues in the LA group showed mild infiltration of immune cells and growth of granulation tissue. Furthermore, severe inflammation and pannus formation in the Pg/LA group were observed (Fig. 1b–e). Inflammation and cartilage damage in joint tissue were more severe in the Pg/LA group compared to other groups (inflammation score 2.75 ± 0.43, cartilage damage score 2.50 ± 0.50) (Fig. 1f, g). The serum ACPA levels and IL-6 levels in serum and joint tissues were elevated in the LA, Pg, and Pg/LA groups compared to the Ctrl group, with the highest levels in the Pg/LA group (Fig. 1h–j). The most elevated level of serum IL-6 in the Pg/LA group among four groups corresponded to our former report that examined the same panel of experiment groups using SKG mouse model [16]. In the present study, although serum IL-6 in the group of LA alone was lower than that of Pg/LA group, there was no significant difference between those two group, contrast to the previous study that detected significant difference in serum IL-6 between corresponding two groups [16] Interestingly, the baseline level of serum IL-6 was also higher than previous study [16]. As the result of the ZAP-70 gene mutation in T cells, arthritis occurs spontaneously without LA [40], while the cellular source of IL-6 in SKG appears to be macrophages [41] which can be affected by the PAMPs released from oral and gut microbiome of the mice. In this present study, the SKG mice were bread in the vivarium of Hiroshima University contrast to the former study that used SKG mice bred in Japan Clea corp [16]., although some discrepancy in the productions of serum IL-6 between present and former of our study using SKG RA mouse model may be attributed to the distinct microbiomes established in the mice at two different facilities.

### Effect of Pg oral administration on gut microbiota composition

**Fig. 2:**
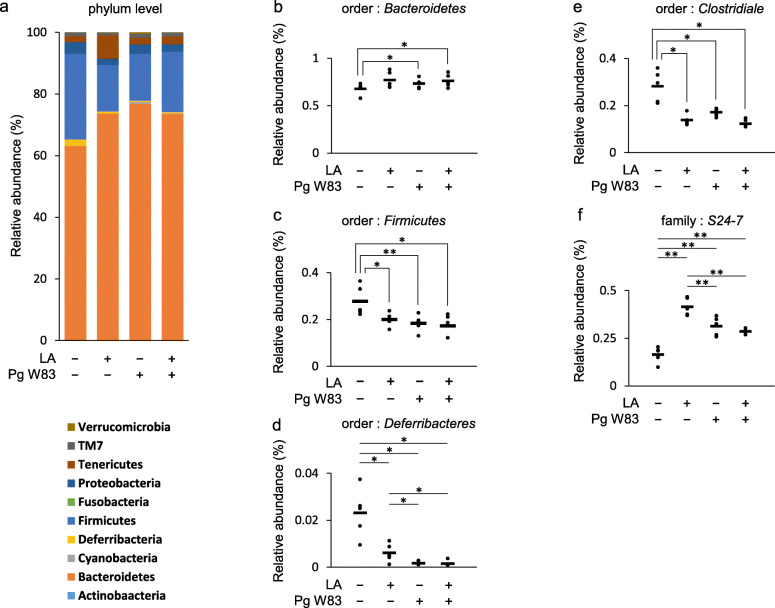
Dysbiosis of gut microbiota with Pg inoculation in SKG mice. Feces from each group were collected for DNA purification. Subsequently, gut microbiota composition was determined by sequencing the bacterial 16S rRNA gene after DNA purification of mouse feces. The abundance of bacterial composition was analyzed. Percentages of phylum-level bacterial composition in each group (**a**). Percentages of order-level or family-level bacterial composition in feces of each group of SKG mice 6 weeks after inoculation with or without Pg (**b–f**). Data represent mean ± SD (**a–f**) of 5 mice per group. Statistical analyses were performed using the Tukey-Kramer test and Bonferroni-corrected Mann-Whitney *U* test for multiple comparisons (*P < 0.05, **P < 0.01)

The composition and abundance of gut microbiota in mouse fecal samples were assessed by next-generation sequencing of 16 s rRNA genes. The relative abundance in phylum level in the LA, Pg, and Pg/LA groups was changed compared to the Ctrl group (Fig. 2a). Further, the LA, Pg, and Pg/LA groups showed increased relative abundance at the order level of Bacteroides compared to controls (Fig. 2b). Conversely, the relative abundance of Firmicutes, Deferribacteres, and Clostridiales was decreased in LA, Pg, and Pg/LA groups compared to Ctrl (Fig. 2c–e). The relative abundance of Deferribacteres in the Pg and Pg/LA groups decreased compared to the LA group (Fig. 2d). At the family level, the relative abundance of S24-7 in the LA, Pg, and Pg/LA groups was significantly increased compared to Ctrl, but the relative abundance of S24-7 in the Pg and Pg/LA groups was lower than that in LA (Fig. 2f).

### Effect of Pg oral administration on gut inflammation

**Fig. 3:**
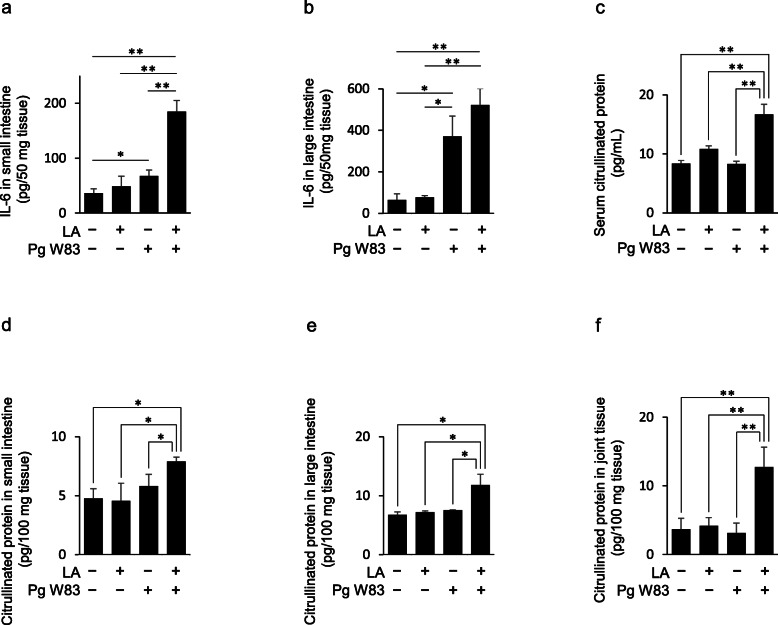
IL-6 production and citrullinated protein levels in intestines and serum. To evaluate intestinal inflammation, IL-6 production in intestinal tissue and serum was measured by ELISA (**a** small intestine, **b** large intestine). To assess CP synthesis, ELISA was performed (**c–f**). Data represent mean ± SD (**a**–**f**) of 6 mice per group. Statistical analyses were performed using the Tukey-Kramer test and Bonferroni-corrected Mann-Whitney *U* test for multiple comparisons (*P < 0.05, **P < 0.01)

To assess gut inflammation, IL-6 production in the tissues of the small and large intestine was measured (Fig. 3a, b). IL-6 production in the small intestine of the Pg/LA group was remarkably elevated compared to other groups (Pg/LA group, 184.8 pg/50 mg tissue). In the Pg group, IL-6 production in the small intestine was slightly increased compared to the Ctrl group (Ctrl group, 36.4 pg/50 mg tissue; Pg group, 67.8 pg/50 mg tissue). Further, IL-6 production in the large intestine of the Pg/LA and Pg groups was remarkably elevated compared to other groups (Pg/LA group, 521.9 pg/50 mg tissue; Pg group, 370.7 pg/50 mg tissue). IL-6 production in the small and large intestine of the LA group was not elevated after 6 weeks. This could be attributed to the induction of experimental arthritis by the single injection of LA takes at least 20 weeks [27]. Thus, at 6 week, it is too early to see the effects of proinflammatory factors produced in arthritis lesion on the level of IL-6 in other tissue, especially in gut, via systemic dissemination.

### Effect of Pg oral administration on CP generation

ACPA-activated inflammation depends on CP generation [11]. Therefore, CP generation in serum and tissues of small and large intestines, and joint was measured by ELISA (Fig. 3c–f). Elevated CP in the Pg/LA group was observed in small intestinal tissue, large intestinal tissue, serum, and joint tissue (1.73-, 1.64-, 3.03-, and 1.65-fold compared to the LA group). CP generation in the small and large intestine of the LA group was not elevated after 6 weeks for the same reason that IL-6 was not elevated in gut tissue, as noted above same reason as the IL-6 elevation in gut tissue.

### Effect of FMT on onset of experimental arthritis

**Fig. 4:**
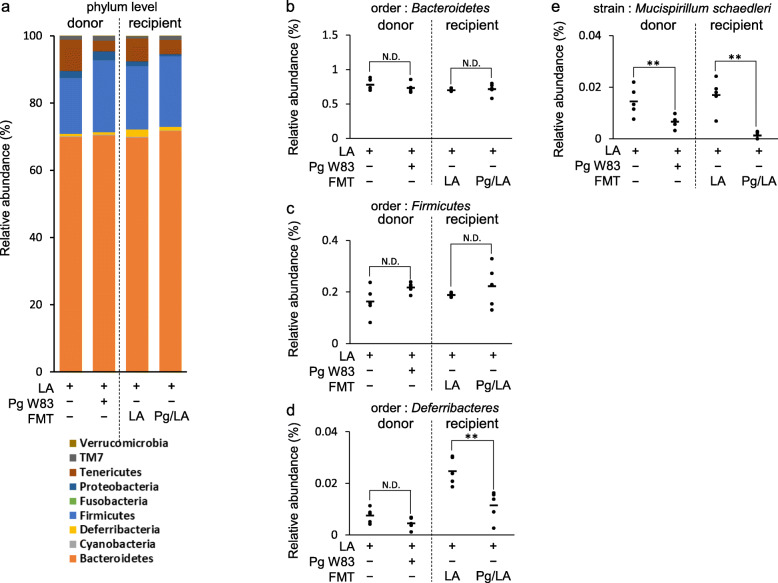
Dysbiosis of gut microbiota in SKG mice. Fecal microbiota transmission (FMT) of donor feces to recipient SKG mice was performed. Recipient mice received donor feces after i.p. injection of LA twice a week. The feces of recipient mice were collected 6 weeks after LA injection, and metagenomics analysis was performed. Percentages of phylum-level bacterial composition in each group of mice (**a**). Percentages of order-level bacterial composition in the feces of each group of SKG mice 6 weeks after FMT (**b–d**). Data represent mean ± SD (**a–d**) of 5 mice per group. Statistical analyses were performed using the Tukey-Kramer test and Bonferroni-corrected Mann-Whitney *U* test for multiple comparisons (*P < 0.05, **P < 0.01)

Pg oral inoculation and LA injection in SKG mice induced RA-like experimental arthritis. Furthermore, Pg inoculation resulted in changes in gut microbiota and inflammation of gut tissue in this model. To determine if gut microbiota change induced by Pg inoculation were important in the exacerbation of experimental arthritis, FMT of feces derived from LA and Pg/LA donor mice was performed on LA-injected recipient mice. Feces were collected from the donor mice (LA and Pg/LA mice: 6 weeks after LA injection with or without Pg inoculation). The feces from recipient mice were collected 6 weeks after the LA injection with FMT. The composition and abundance of gut microbiota in mouse fecal samples from donor mice and recipient mice were assessed by percentages of order-level and phylum-level bacterial composition (Fig. 4a–d). Composition at the phylum level of LA-FMT and Pg/LA-FMT recipient groups was similar to that of the LA and Pg/LA donor groups, respectively. Although the relative abundance of Bacteroides or Firmicutes showed the difference between sham group and Pg oral inoculated group in C57BL/6 mice, no differences were observed in the relative abundance of Bacteroides or Firmicutes in donor group as well as recipient groups (Fig. 4b, c) [42]. On the other hand, contrast to Bacteroides and Firmicutes, the relative abundance of Deferribacteres in the gut microbiome of LA-treated recipient group showed significant decrease by FMT from Pg/LA group, compared to that received FMT from LA group (Fig. 4d). While there was no statistically significant difference (*P* = 0.103), LA-treated donor groups showed the similar trend of distribution in relative abundance of Deferribacteres to that found in the FMT recipient mice (Fig. 4d). It is noteworthy that, among Deferribacteres, *Mucispirillum schaedleri* (also called ASF 457), which represents a commensal bacterium in altered Schaedler flora (ASF) of mice [43, 44], showed the significant decrease in both donor and recipient groups in a Pg-dependent manner (Fig. 4e). These results indicated that oral inoculation of Pg can affect the gut microbiome, which was represented by the suppression of Deferribacteres abundance, and such trait was also carried over to the FMT recipient mice. As a control of FMT, the buffer used for fecal suspension was inoculated into mice after the LA injection (the control group for the FMT experiment). The microbiome between the LA injected group (donor group) and the control group for the FMT experiment with LA injection did not show the statistical difference (data not shown).

**Fig. 5:**
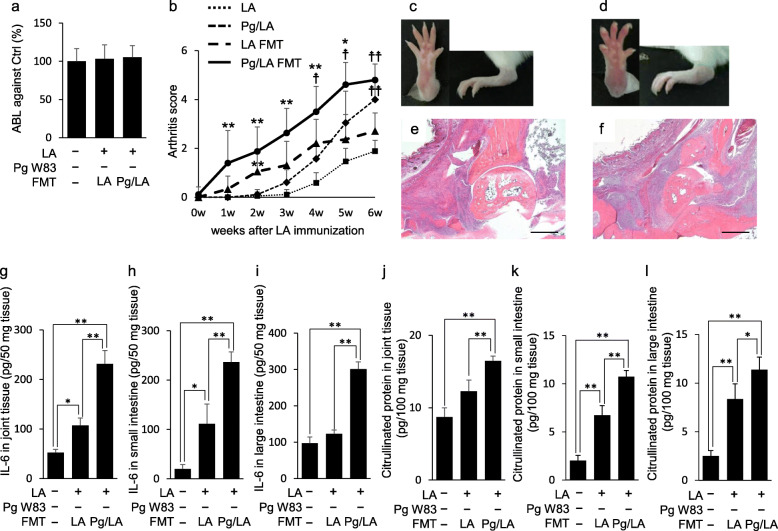
Effect of FMT in SKG mice. The effect of FMT on alveolar bone destruction was determined. In this experiment, Pg W83 strain was used. ABL of the upper jaw in each group was measured (**a**). AS was measured every week after FMT and LA i.p. injection (**b**). Representative figures of hind paws in four groups showing joint inflammation (**c** LA-FMT, **d** Pg/LA-FMT). H&E staining of joint tissue sections (**e** LA-FMT, **f** Pg/LA-FMT). Original magnification; scale bar 500 μm. To evaluate intestinal inflammation 6 weeks after FMT, IL-6 production in joint and intestinal tissues of SKG mice was measured by ELISA (**g**–**i**). To assess CP synthesis, ELISA was performed in the joint tissues (**j**), small intestines (**k**), and large intestines (**l**). Data represent mean ± SD (**b**, **c**, and **h**–**m**) of 6 mice per group. Statistical analyses were performed using the Tukey-Kramer test and Bonferroni-corrected Mann-Whitney *U* test for multiple comparisons (*P < 0.05, **P < 0.01)

To further assess the effect of FMT on the onset of experimental arthritis, mouse periodontal and joint tissues were analyzed. FMT of donor feces to the recipient mouse was performed through gastric intubation. Therefore, it is conceivable that FMT might have no effect on oral microbiota. To confirm the absence of FMT’s effect on oral cavity, alveolar bone healthy mice were morphologically evaluated. FMT did not show any effect on alveolar bone (Figs. S2 and 5a), suggesting that FMT has little or no effect on oral microbiome that may cause pathogenic bone resorption. However, severe joint swelling was observed in the LA-FMT and Pg/LA-FMT groups compared to the LA and Pg/LA groups (Fig. 5b–f, Fig. 1c, e). The maximum AS of the LA-FMT and Pg/LA-FMT groups was much higher than that of LA and Pg/LA, respectively. Pannus formation in the Pg/LA-FMT group was found in joint tissues, similar to that found in the Pg/LA group. Pannus formation and erosion of joint bone tissues were strongly observed in the LA-FMT and Pg/LA-FMT groups (Fig. 5e, f). Additionally, FMT of feces from the LA and Pg/LA groups increased IL-6 in the tissues of joint, small intestine, and large intestine (Fig. 5g–i). CP levels in the tissues of joint, small intestine, and large intestine were also elevated in the FMT-LA and FMT-Pg/LA groups, with the Pg/LA group showing the highest levels (Fig. 5j–l). The FMT of the feces from the Pg/LA group showed no joint swelling in the absence of LA injection. Similarly, FMT of feces from the Pg group did not show severe arthritis compared with FMT of feces from the LA and Pg/LA groups in the presence of LA injection (data not shown).

### Effect of PgPAD-deficient mutant on exacerbation of experimental arthritis

**Fig. 6:**
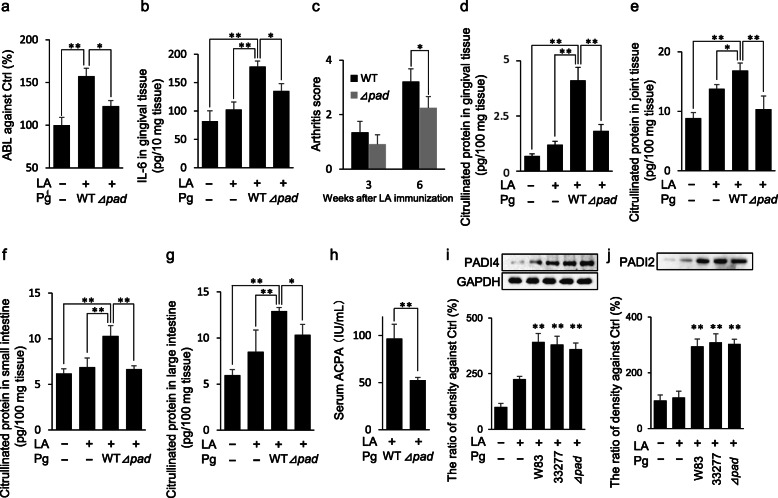
Effect of PgPAD on the onset of joint arthritis. The effect of PgPAD on the onset of arthritis was determined. In this experiment, PgPAD deletion mutant 33277 strain (⊿*pad*) and Pg 33277 wild-type (WT) strain were used, unless Pg W83 strain was specifically described in caption of Figures. ABL of the upper jaw in each group was measured (**a**). Ctrl, PBS inoculation; WT, Pg wild-type inoculation; ⊿*pad*, Pg *pad* knockout strain inoculation. To evaluate inflammation of the gingival tissue 6 weeks after Pg inoculation, IL-6 production in gingival tissue of SKG mice was measured by ELISA (**b**). AS was measured 3 and 6 weeks after Pg inoculation with LA i.p. injection (**c**). To assess CP synthesis, ELISA was performed (**d** gingival tissue, **e** joint tissue, **f** small intestine, **g** large intestine). The titer of serum ACPA 6 weeks after Pg inoculation with LA i.p. injection in mice from each group was measured by ELISA (**h**). The production of endogenous PADI2 and PADI4 in joint tissue was determined by Western blotting, and then the density of each band was measured by NIH Image-J (**i** and **j**). Data represent mean ± SD (**b**–**j**) of 5 mice per group. Statistical analyses were performed using the Tukey-Kramer test and Bonferroni-corrected Mann-Whitney *U* test for multiple comparisons (*P < 0.05, **P < 0.01)

To determine the effect of PgPAD on CP generation and exacerbation of experimental arthritis, the PgPAD-deficient Pg 33277 strain as well as Pg 33277 wild type was inoculated into the oral cavity of SKG mice. Bone resorption around the upper molar teeth of Pg 33277 wild-type strain and PgPAD deficient Pg strain was measured (Fig. S3a). Oral inoculation of the PgPAD-deficient Pg strain showed slight bone resorption compared to the Ctrl group, which was much lower than that of the wild-type strain (35.2% lower, Fig. 6a). IL-6 production in gingival tissue of the wild type-inoculated group was elevated. However, this increase was suppressed in the deficient strain (Fig. 6b). Additionally, AS of the PgPAD-deficient Pg strain was significantly lower than the wild-type Pg 33277 strain (Fig. 6c). CP generation in serum and tissues of joint, small intestine, and large intestine by inoculation of the PgPAD-deficient Pg strain was also lower than that of the wild-type strain (55.6%, 38.2%, 35.9%, and 20.2% decrease, respectively, Fig. 6d–g). Serum ACPA levels in the PgPAD-deficient Pg strain were significantly decreased compared to \wild-type (45.8% decrease, Fig. 6h). The induced production of IL-6 in gingival tissue, CP generation in gingival tissue, and CP generation in large intestine by the inoculation of Pg 33277 wild-type strain was suppressed by the inoculation of PgPAD-deficient Pg 33277 strain. In order to confirm the effect of PgPAD on the progression of arthritis, the internal PAD (PADI2 and PADI4) production in mouse joint tissue was determined. The inoculation of Pg W83, Pg 33277 wild-type, and Pg 33277 PgPAD-deficient strain did not show any difference in the production of PADI2 and PADI4 (Fig. 6i, j). Pg infection in mouse gingival tissue was also confirmed by detection of Pg protein in the gingival tissue homogenates using anti-Pg specific Western blotting (Fig. S3b). In summary, these results using Pg 33277 PgPAD-deficient strain and Pg 33277 wild type demonstrated that Pg PAD is engaged in elevations of citrullinated protein and ACPA without affecting the productions of host’s endogenous PADI1 and PADI4, suggesting that PgPAD is responsible for the increased pathogenic outcomes of RA by elevated productions of citrullinated protein and ACPA.

## Discussion

In this study, Pg oral inoculation induced experimental arthritis in SKG mice by altering gut microbiota. FMT from the feces of Pg-inoculated arthritis mice to uninoculated mice caused joint destruction. Concomitantly, CP levels in intestinal and joint tissues were elevated, but partially suppressed in the absence of PgPAD in the PgPAD-deleted mutant group.

Previous reports showed that periodontitis affected gut microbiota [45]. The inoculation of periodontopathogenic bacteria resulted in the change of gut microbiota and this change induced the destruction of tight junction in the ileum [46]. Human Microbiome Project by genome biology showed that oral cavity and stool bacteria overlapped in nearly 45% of the subjects [47].

Clinical studies also demonstrated the effect of periodontopathogenic bacteria on gut microbiota [45], likely from the daily inoculation of 1–2 L of saliva, including pathogenic bacteria (approximately 10^9^ CFU/mL) [24]. While planktonic Pg can be eliminated at low pH (pH 5), the biofilm of inoculated Pg can survive in acidic conditions as low as artificial gastric juice (pH 3) [25].

In this study, Pg inoculation affected gut microbiota composition. Pg inoculation resulted in decreased relative abundance of Deferribacteres and S24-7 in Pg/LA compared to the LA group (Fig. 2d, f). By contrast, the abundance of Bacteroides, Firmicutes, and Clostridiales did not show significant differences among groups, although their relative abundance changed dramatically compared to the control group (Fig. 2). Previous reports showed that Pg administration in the DBA/1 J experimental arthritis mouse model resulted in decreased relative abundance of Bacteroidetes (phylum level) and Bacteroides (genus level), but increased Firmicutes (phylum level) [25]. In other systemic disease models (type 2 DM in C57BL/6 mice), Pg inoculation increased the abundance of Bacteroides and decreased Firmicutes [46].

However, the administered Pg DNA fragment and protein were not detected in intestinal feces of the Pg and Pg/LA groups (data not shown). In our previous study, *Escherichia coli* stimulation did not affect the degree of experimental arthritis, as compared to arthritis without *E*. *coli* stimulation [15]. Another periodontopathogenic bacterium *Prevotella intermedia* did not show any differences compared to experimental arthritis without bacterial stimulation [25]. In humans, *Prevotella copri* was isolated from RA patient samples and identified as a key bacterium [48] involved in arthritis progression. However, in our mouse model, neither *P*. *copri* nor genetically close bacteria were detected in feces of the arthritis-induced model (data not shown). However, after 6 weeks from oral administration of Pg, the administered Pg DNA fragment was not detected in intestinal tissues and feces of the Pg and Pg/LA groups. In the purified DNA from serum, feces, gingival tissue, tongue, lung, stomach, small intestine, and large intestine did address to detect Pg- and *P*. *copri*-derived DNA fragment by specific primer to 16 s rRNA. Pg-derived DNA was only detected from gingival tissue, tongue, and lung tissue (Fig. S1d). This is because the bacteria inoculated in this study were the planktonic condition. Therefore, the way of Pg biofilm inoculation should be performed in the future study.

FMT-mediated alteration of gut microbiota composition has been established to treat gastrointestinal disorders, such as Crohn’s disease and ulcerative colitis [49]. In FMT-treated patients, an increase in bacterial diversity and abundance of Bacteroides and Firmicutes was observed, along with improved of clinical symptoms. In the mouse model, fecal transplantation from RA patients to mice replicated the human gut microbiota of RA and the onset of experimental arthritis via intestinal Th17 cell activation [22]. In the present study, the FMT from RA-induced donor mice that were orally colonized with Pg, but not control RA-induced donor mice, caused the alteration of gut microbiota in recipient RA-induced mice which, in turn, induced gut inflammation and CP generation, thereby exacerbating arthritis (Figs. S2 and 5). While elevation of CP generation in the mice received Pg oral inoculation was mediated by PgPAD in a manner independent of host’s endogenous PADIs (Fig. 6), FMT that did not contain any Pg could also promote the CP generation in gut and RA tissue, indicating that dysbiosis of gut microbiota appeared to act on host’s endogenous PADIs. Despite limitations of comparing differences between human and mouse microbiota, these studies highlight the importance of gut dysbiosis in the progression of systemic disease, including RA, in the absence of pathogenic oral Pg in gut microbial flora. Both periodontitis and RA treatments resulted in changes in the gut microbiota [45]. Therefore, the monitoring of gut microbiota and specific bacterial species during the treatment of both periodontitis and RA would be useful for identifying risk factors, determining prognosis, and administering therapeutics.

ACPAs target proteins/peptides with citrullinated epitopes and serve as informative RA biomarkers, which are useful for RA diagnosis [50]. ACPAs are generated within synovium and possibly at extra-articular sites prior to disease onset. Recent investigations have begun to elucidate the different mechanisms by which ACPAs may be directly pathogenic in RA. CP is a specific target of ACPA and involved in ACPA generation. This study found increased CP in gingiva, small intestine, large intestine, and joint tissues following Pg inoculation in the experimental arthritis model mouse (Figs. 3, S3, and 6). In humans, four citrullinated autoantigens, including fibrinogen/fibrin, vimentin, α-enolase, and type II collagen, are now well accepted as ACPA targets [51–54]. In our study, the specific origin of CP is unclear. However, Pg can rapidly generate CP from α-enolase or fibrinogen by proteolytic cleavage at Arg-X peptide bonds using arginine gingipains, followed by citrullination of carboxyterminal arginines by bacterial PAD [13]. Taken together, one of these mechanisms may lead to ACPA generation by PgPAD citrullination. To support this hypothesis, the Pg knockout mutant of PgPAD resulted in less CP generation in serum and in gingival, small and large intestine, and joint tissues (Fig. 6d–g). Furthermore, Pg 33277 wild type-stimulated serum ACPA was diminished by stimulation with the PgPAD knockout mutant (Fig. 6h). However, PADI4, an endogenous PAD, is also involved in CP and ACPA generations, according to the report demonstrating that PADI4 knockout mice decrease severity of experimental autoimmune arthritis [55]. The induction of PADI4 is mediated by TNF-α, IL-6, and neutrophil extracellular traps (NETs, NETosis) [55–57]. Increased CP in joint tissue resulted in increased synovial fibroblast migration and spread ability [58]. In lung tissues, CP synthesis and CP-induced inflammation were also reported [59]. Pg inoculation has been shown to induce inflammatory responses in periodontal tissues and in local and systemic sites. In our model, Pg infection activated IL-6 production in the small intestine, large intestine, and joint tissue (Figs. 1i, j, and 3a, b), resulting in PADI4 induction and subsequent CP and ACPA generation.

Surprisingly, PgPAD knockout showed both decreased CP and ACPA production and suppressed inflammation of periodontal tissues, including alveolar bone resorption (Fig. S3a and 6a), which depends on osteoclast activation via RANKL signaling [60]. A previous report showed that citrullinated vimentin induced endogenous PADI4 and RANKL in fibroblast-like synoviocytes derived from RA patients [61]. However, no previous study has shown a correlation between PgPAD and inflammation. Yet, the combination of gingipain and PgPAD generated citrullinated α-enolase, and its CP might be an initiator of inflammation. In this present study, Pg 33277 wild type and PgPAD knockout mutant were used. The effects of Pg 33277 on the production of IL-6 and arthritis score showed similar trends to those of Pg W83 (Figs. 1 and 6), suggesting that both stains of Pg can augment the pathogenic outcomes of RA. Importantly, although the production of internal PADI2 and PADI4 level showed no difference among Pg W83, Pg33277, and PgPAD mutant (Fig. 6i, j), the amounts of Pg recovered in gingival tissue among these Pg strains were comparable (Fig. S3b). Therefore, PgPAD has the potential for induction of arthritis. There was a report using PgPAD knockout mutant in the ACPA generation in a mouse model. In that study, alveolar bone resorption, joint swelling, and ACPA production corresponded to our result [62].

## Conclusions

Based on our findings, we propose the following working hypothesis of arthritis pathogenesis with Pg inoculation (administration) in SKG mice: (1) continued Pg inoculation results in changes in the gut microbiota, (2) dysbiosis of gut microbiota induces inflammation in intestinal tissues, (3) increased CP in the intestine accelerates systemic ACPA production, (4) severe joint destruction, and (5) CP generation via PgPAD. However, arthritis was also found in the PgPAD knockout mutant, and its AS was higher than that of the normal arthritis model, indicating that Pg is an induction factor for arthritis exacerbation. Therefore, further study is needed to clarify the molecular mechanisms underlying the involvement of Pg infection in the onset of RA.

## Supplementary information

**Additional file 1: Figure S1.** Assessment of periodontal tissue and evaluation of joint swelling in Pg-administered SKG mice. To determine the effect of Pg infection on periodontal tissue, Pg W83 (10^8^ CFU/50 mL/mouse in 2% CMC/PBS solution) was administered into the oral cavity of SKG mice (6–8 weeks old, female) twice a week for 42 days. SKG mice also received LA i.p. injection. All mice were sacrificed, and gingival and leg joint tissues were collected. Morphological observation of the mouse upper jaw was analyzed (a). ABL of the upper jaw in each group was measured (b). Joint swelling was quantified using Sakaguchi’s AS every week after oral inoculation of Pg (1.0 × 10^8^ CFU/mouse) and LA i.p. injection (c). Ctrl: PBS inoculation, LA: PBS inoculation + LA i.p. injection (10 μg/mouse), Pg: Pg inoculation, Pg/LA: Pg inoculation + LA i.p. injection. Data represent mean ± SD (b, c) of 6 mice per group. Data represent mean ± SD (b, c) of 6 mice per group. Statistical analyses were performed using the Tukey-Kramer test and Bonferroni corrected Mann-Whitney *U* test for multiple comparisons (* *P* < 0.05, ** *P* < 0.01). After 6 weeks from oral administration of Pg, Pg-derived DNA fragment by specific primer to 16 s rRNA in the purified DNA from serum, feces, gingival tissue, tongue, lung, stomach, small intestine, and large intestine (d). **Figure S2.** Effect of FMT in SKG mice. The effect of FMT on mouse periodontal tissue was determined by monitoring of periodontal bone. Morphological observation of the periodontal alveolar bone of upper jaw of healthy mice was evaluated after 42 days from FMT. Ctrl: PBS inoculation, LA FMT: FMT feces from LA mouse + LA i.p. injection (10 μg/mouse), FMT Pg/LA: FMT feces from Pg/LA mouse + LA i.p. injection. **Figure S3.** Effect of PgPAD on the onset of joint arthritis. The effect of PgPAD on the onset of arthritis was determined (a). Morphological observation of the mouse upper jaw was analyzed. Ctrl: PBS inoculation, Pg WT/LA: Pg wild type inoculation + LA i.p. injection, Pg ⊿*pad*/LA: Pg *pad* knockout strain inoculation + LA i.p. injection. The amount of infected Pg was determined by Western blotting (b). Positive control showed 10^5^ CFU Pg W83 lysate per well.

## Data Availability

The datasets used and/or analyzed during the current study are available from the corresponding author on reasonable request.

## Abbreviations

ABL: Alveolar bone loss
AS: Arthritis score
CMC: Carboxymethylcellulose
EDTA: Ethylene diamine tetrameric acid
HE: Hematoxylin and eosin
i.p.: Intraperitoneal
LA: Laminarin
Micro-CT: Micro-computed tomography
PD: Periodontal disease
RA: Rheumatoid arthritis
SEM: Standard error of the mean
sRANKL: Soluble recombinant receptor activation of nuclear factor kappa-B ligand
