# Supplementary material for: Effect of Porphyromonas gingivalis infection on gut dysbiosis and resultant arthritis exacerbation in mouse model
Source: Arthritis Res Ther. 2020 Oct 19;22:249. doi: 10.1186/s13075-020-02348-z (PMC7574451; doi:10.1186/s13075-020-02348-z)
Supplement: Supplementary file 1 — Additional file 1: Figure S1. Assessment of periodontal tissue and evaluation of joint swelling in Pg-administered SKG mice. To determine the effect of Pg infection on periodontal tissue, Pg W83 (108 CFU/50 mL/mouse in 2% CMC/PBS solution) was administered into the oral cavity of SKG mice (6–8 weeks old, female) twice a week for 42 days. SKG mice also received LA i.p. injection. All mice were sacrificed, and gingival and leg joint tissues were collected. Morphological observation of the mouse upper jaw was analyzed (a). ABL of the upper jaw in each group was measured (b). Joint swelling was quantified using Sakaguchi’s AS every week after oral inoculation of Pg (1.0 × 10^8^ CFU/mouse) and LA i.p. injection (c). Ctrl: PBS inoculation, LA: PBS inoculation + LA i.p. injection (10 μg/mouse), Pg: Pg inoculation, Pg/LA: Pg inoculation + LA i.p. injection. Data represent mean ± SD (b, c) of 6 mice per group. Data represent mean ± SD (b, c) of 6 mice per group. Statistical analyses were performed using the Tukey-Kramer test and Bonferroni corrected Mann-Whitney U test for multiple comparisons (* P < 0.05, ** P < 0.01). After 6 weeks from oral administration of Pg, Pg-derived DNA fragment by specific primer to 16 s rRNA in the purified DNA from serum, feces, gingival tissue, tongue, lung, stomach, small intestine, and large intestine (d). Figure S2. Effect of FMT in SKG mice. The effect of FMT on mouse periodontal tissue was determined by monitoring of periodontal bone. Morphological observation of the periodontal alveolar bone of upper jaw of healthy mice was evaluated after 42 days from FMT. Ctrl: PBS inoculation, LA FMT: FMT feces from LA mouse + LA i.p. injection (10 μg/mouse), FMT Pg/LA: FMT feces from Pg/LA mouse + LA i.p. injection. Figure S3. Effect of PgPAD on the onset of joint arthritis. The effect of PgPAD on the onset of arthritis was determined (a). Morphological observation of the mouse upper jaw was analyzed. Ctrl: PBS inoculation, Pg WT/LA: Pg wild type inocul [file 13075_2020_2348_MOESM1_ESM.pptx]

## Slide 1
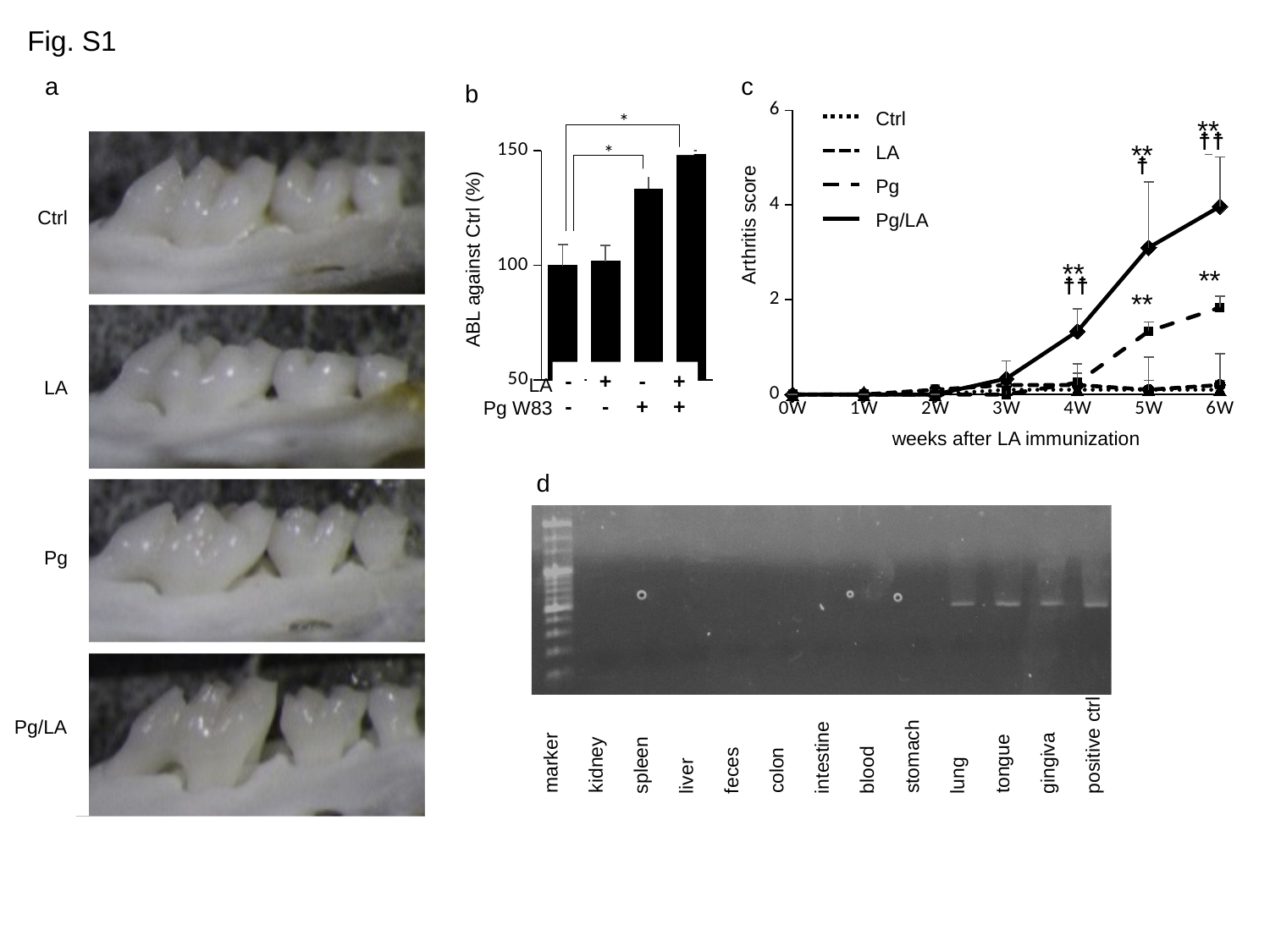

Fig. S1
a
c
b
### Chart
| Category | C | LA | Pg | LA/W83 |
|---|---|---|---|---|
| 0W | 0.0 | 0.0 | 0.0 | 0.0 |
| 1W | 0.0 | 0.0 | 0.0 | 0.0 |
| 2W | 0.0 | 0.0 | 0.1 | 0.0 |
| 3W | 0.1 | 0.0 | 0.2 | 0.3333333333333333 |
| 4W | 0.1 | 0.25 | 0.2 | 1.3333333333333333 |
| 5W | 0.1 | 1.3333333333333333 | 0.1 | 3.1 |
| 6W | 0.1 | 1.833333333333333 | 0.2 | 3.966666666666667 |Ctrl
*
**
☨☨
**
*
LA
### Chart
| Category | |
|---|---|
| Ctrl | 100.0 |
| LA | 101.85185185185183 |
| Pg | 133.33333333333331 |
| PL | 148.14814814814815 |☨
Pg
Ctrl
Pg/LA
Arthritis score
ABL against Ctrl (%)
**
**
☨☨
**
-
-
+
-
-
+
+
+
LA
Pg W83
LA
weeks after LA immunization
d
positive ctrl
stomach
marker
lung
kidney
blood
gingiva
intestine
tongue
colon
spleen
feces
liver
Pg
Pg/LA

## Slide 2
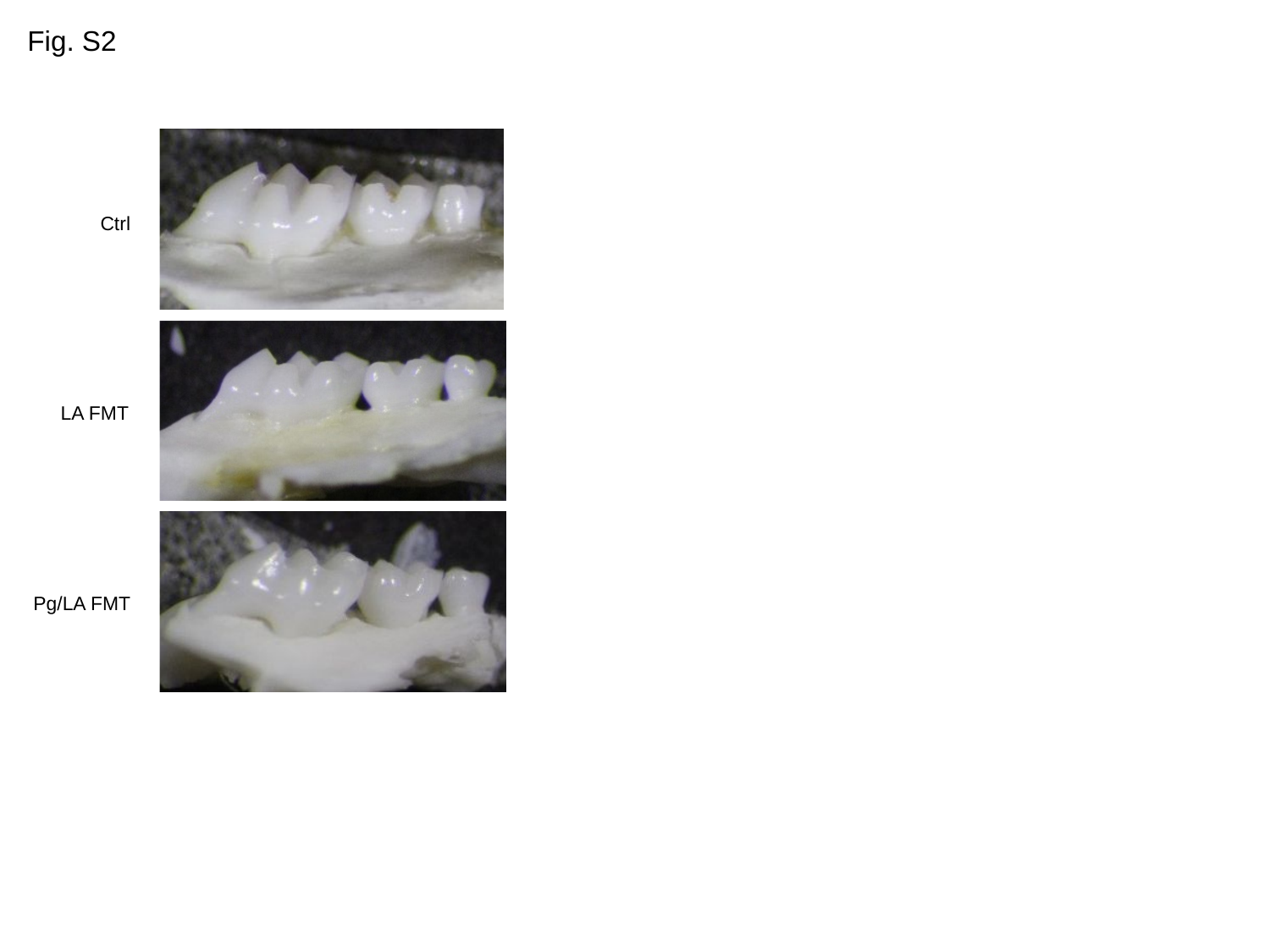

Fig. S2
Ctrl
LA FMT
Pg/LA FMT

## Slide 3
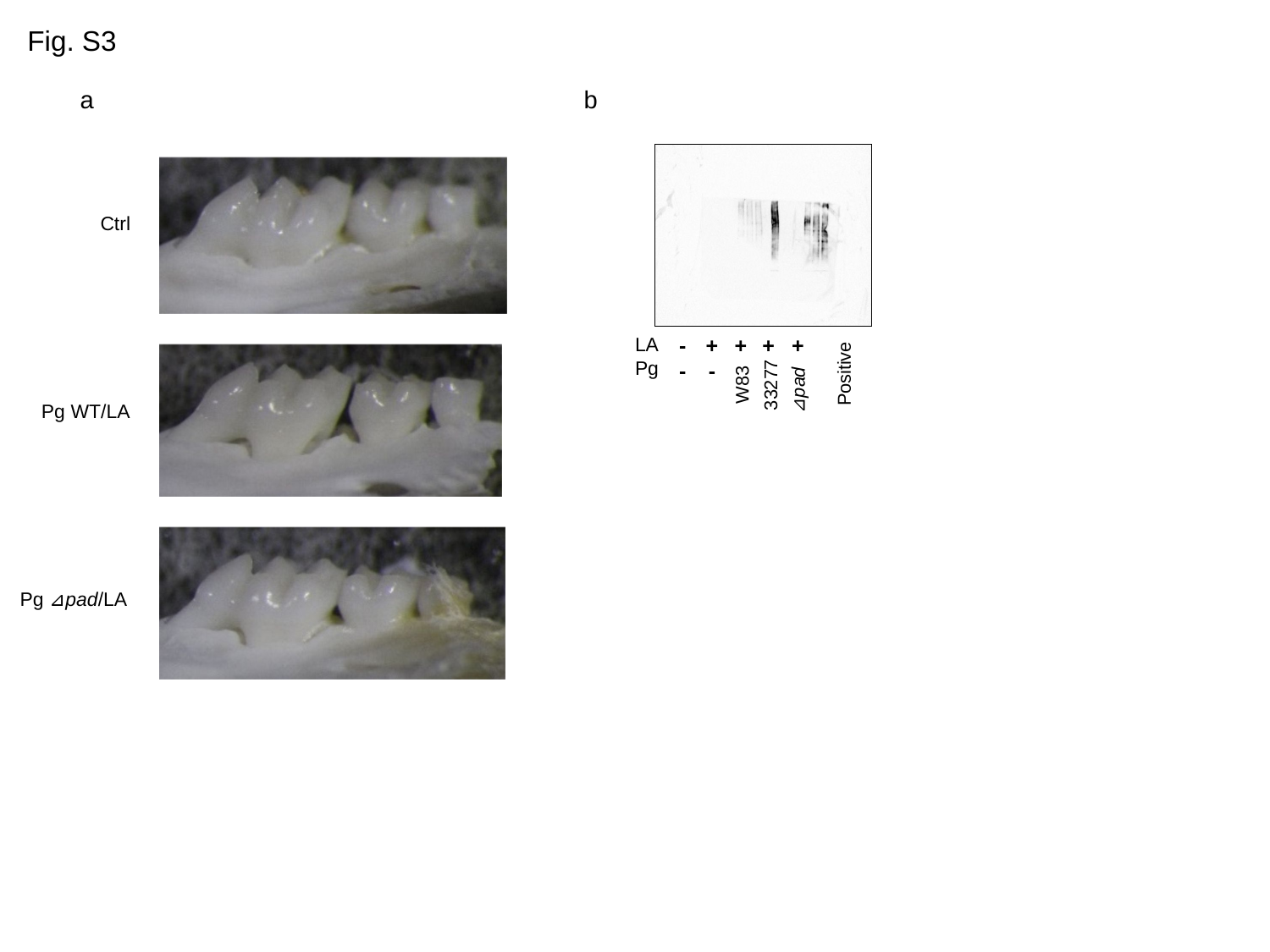

Fig. S3
b
a
Ctrl
LA
Pg
-
-
+
-
+
+
+
Positive
W83
33277
⊿pad
Pg WT/LA
Pg ⊿pad/LA
